# Development and Comprehensive Diverse-Matrix Evaluation of Four PAHs Using Solvent-Modified QuEChERS-GC-MS with Freeze-Out

**DOI:** 10.3390/foods14172979

**Published:** 2025-08-26

**Authors:** Kyung-Jik Lim, Hyun-Jun Kim, Yu-Jin Heo, Han-Seung Shin

**Affiliations:** Department of Food Science and Biotechnology, Dongguk University-Seoul, 32, Dongguk-ro, Ilsandong-gu, Goyang-si 10326, Gyeonggi-do, Republic of Korea; kyung9209@naver.com (K.-J.L.); tellingtime@naver.com (H.-J.K.); pdp0616@naver.com (Y.-J.H.)

**Keywords:** polycyclic aromatic hydrocarbon, QuEChERS method, gas chromatography-mass spectrometry, food analysis, dietary exposure

## Abstract

Polycyclic aromatic hydrocarbons (PAHs) are recognized carcinogens that enter the food chain through pre-existing environmental contamination (air, water, soil), and their formation and accumulation during food preparation and processing involve high temperatures. We established a modified QuEChERS GC-MS method that couples *n*-hexane-saturated acetonitrile containing 1% toluene with a freeze-out step. Compared to the previously reported ACN QuEChERS protocol, this method enhanced PAH desorption and suppressed lipid interference across four matrices. The method linearity (R^2^ ≥ 0.99), limit of detection (LOD, from 0.03 to 0.20 μg/kg), limit of quantitation (LOQ, from 0.10 to 0.60 μg/kg), and intra-/inter-day precision (≤5.7% RSD) all satisfied AOAC criteria. The modified QuEChERS reduced solvent consumption and shortened preparation time compared to other conventional extraction methods. The developed method was applied to 302 retail food samples, and *Kezuribushi* was found to have the highest concentration of the 4PAHs, reaching 22.0 µg/kg. Risk assessment based on EFSA’s margin-of-exposure (MOE) approach identified grilled chicken feet (MOE = 7604) as a potential health concern, as this value falls below EFSA’s threshold of 10,000 for potential risk characterization. The validated method enables sensitive and scalable monitoring of PAHs in complex food matrices within the tested matrices and conditions.

## 1. Introduction

Polycyclic aromatic hydrocarbons (PAHs) are aromatic hydrocarbon compounds consisting of multiple fused benzene rings, and they originate primarily from the incomplete combustion or thermal degradation of organic matter. They are toxic substances that may contaminate foods, mainly through environmental exposure (air, water, soil) or preparation and processing involving high temperatures (e.g., roasting, frying, grilling) [1,2]. Previous studies have shown that PAHs disperse through air, water, soil, and plants, contaminating aquatic and terrestrial species and subsequently entering the human food supply. Dietary intake accounts for about 96.2% of total exposure, while air, water, and soil contribute only 1.6%, 0.1%, and 1.9%, respectively, confirming that food is the main source [3]. PAHs are non-polar compounds, exhibiting low solubility in water and pronounced hydrophobicity [4]. These molecular properties increase as molecular weight increases [5]. Thermal degradation of endogenous hydrocarbons, combustion of cooking fuels, and pyrolysis of lipid droplets on heat sources are recognized as the primary pathways contributing to PAH generation during food processing [2,6].

As a common convention, PAHs are grouped into two categories based on the number of fused benzene rings in their structure: those containing four or fewer rings are referred to as light PAHs, while those with five or more rings are categorized as heavy PAHs [7]. The carcinogenicity and tumorigenic potential of these compounds have been demonstrated in animal studies and epidemiological cohort investigations [8]. Codex CXC 68-2009 provides guidance to reduce PAH exposure, such as replacing direct smoking with indirect smoking where feasible, prohibiting the use of high-PAH fuels, cleaning smoke streams by filtration or tar removal, and implementing process controls to minimize contamination [9]. The International Agency for Research on Cancer (IARC) has classified benzo[a]pyrene (BaP) as a Group 1 carcinogen [10]. This classification indicates sufficient evidence of carcinogenicity in humans. Benz[a]anthracene (BaA), chrysene (CHR), and benzo[b]fluoranthene (BbF) are categorized as Group 2A or 2B substances, suggesting probable or possible carcinogenicity.

In December 2002, the Scientific Committee on Food (SCF) of the European Union (EU) identified 15 high-molecular-weight PAHs as compounds with genotoxic and carcinogenic properties [11]. In February 2005, the European Commission emphasized the need for analytical monitoring of these compounds in foods, in response to concerns regarding human exposure to PAHs through dietary intake and the associated health risks [12]. Based on this, the European Food Safety Authority (EFSA) issued an official opinion proposing four marker compounds—BaP, BaA, BbF, and CHR (4PAHs)—as key indicators for evaluating PAH contamination in food [13].

In response to PAH contamination in food, the EU has established maximum limits for various food categories since 2011. Key regulated products included smoked foods, vegetable oils, and infant foods, with BaP/Σ4PAHs limits of 5.0/30.0, 2.0/10.0, and 1.0/1.0 µg/kg, respectively [14]. However, previous research has reported that traditionally smoked fish products exceeded regulatory limits, with BaP levels reaching up to 45.0 µg/kg and up to 180.0 µg/kg for Σ4PAHs [15]. Monitoring is being conducted continuously, but because exceedance cases are still found, additional follow-up investigations are required. Accordingly, it is necessary to establish appropriate regulations and maintain continuous control over high-risk food categories to reduce PAH contamination.

In addition to setting maximum limits, the EU has also established specific analytical performance criteria to support the accurate monitoring of PAHs. Regulation (EC) No 333/2007 provides official sampling procedures and outlines method performance requirements such as limits of quantification, recovery ranges, and measurement uncertainty for the determination of BaP in foodstuffs [16]. Later, Regulation (EU) No 836/2011 expanded these provisions to include the four EFSA-recommended marker PAHs, thereby harmonizing control measures for the entire Σ4PAHs group [17]. More recently, Regulation (EU) 2023/915, in force since May 2023, has consolidated and updated the EU contaminants framework by setting maximum levels for both BaP and Σ4PAHs across a wide range of foods, including smoked meats, vegetable oils, cereals, and infant formula, while reinforcing the requirement to meet the analytical criteria established in the earlier regulations European Commission [18].

Quantitative analysis of various PAHs has been conducted in the field of food science for continuous monitoring of PAHs. For high-fat food matrices, liquid–liquid extraction (LLE) is commonly used for sample preparation. This method is recognized by several international and national standards, including ISO 15753:2016—a standard also adopted by the EU—as well as GB 5009.265–2021 (China) and the Ministry of Food and Drug Safety (MFDS) Notice No. 2024-71 (Korea) [19,20,21]. However, the conventional LLE method consumes over 300 mL of organic solvents and requires 6–24 h of sample preparation with complex handling steps. These factors may lead to extended preparation times, sample contamination, and reduced reproducibility. This limits its efficiency in analytical applications [22,23]. Therefore, the QuEChERS (Quick, Easy, Cheap, Effective, Rugged, and Safe) method is considered a suitable alternative for the quantitative analysis of PAHs [24]. QuEChERS is designed to perform extraction, dehydration, and clean-up in a simple, step-by-step process. As a result, the total preparation time can be reduced to less than 30 min, and the use of organic solvents is minimized to 10 mL of acetonitrile (ACN) [24]. The method also enables simultaneous processing of multiple samples.

In food matrices, various factors exist that reduce PAH recoveries, one of which is lipid interference in high-fat samples, and another is PAH adsorption onto carbonized residues formed during high-temperature processing [6,25]. In high-fat samples, studies have reported decreased PAH recoveries when applying the QuEChERS method, highlighting the need for complementary extraction strategies to improve recoveries while preserving its analytical efficiency [26,27]. A previous study has applied an additional freeze-out (Fo) clean-up step to reduce lipid interference and improve recovery in oily samples [28]. Fo preparation alleviates lipid interference by promoting the physical solidification of fat components at low temperatures. In the case of surface carbonization caused by high-temperature cooking, the reduced recoveries are mainly due to different mechanisms. During surface carbonization of food samples, thermal reactions promote the formation of carbonaceous matrices [29]. These matrices strongly undergo π–π stacking interactions with PAHs, thereby contributing to a marked reduction in recovery [30]. Consequently, because low recovery rates have been consistently reported in high-fat and thermally processed food matrices, these findings indicate the necessity of further improvements in the current analytical methods [26].

In this study, an improved analytical method was developed by modifying the conventional QuEChERS protocol. To our knowledge, unlike previous modifications, which have typically applied either extraction solvents or additional lipid removal steps independently, our method combines toluene-modified *n*-hexane-saturated ACN (HA) with Fo step into an integrated method. This method was designed to enhance the extraction efficiency of PAHs by weakening the π–π stacking interactions between carbonized food surfaces and PAHs using the aromatic solvent toluene. Furthermore, lipid interference was minimized by using HA, and a Fo sample preparation technique was applied under optimized conditions to reduce lipid effects further and improve extraction efficiency. Using this approach, an optimized analytical method was established for four representative food matrices, providing a reliable quantification system for 4PAHs. The developed validated method was applied to 302 food samples collected across Korea to evaluate 4PAHs concentrations. Dietary exposure levels were estimated based on 74 food items with complete analytical data. The findings of this study are expected to contribute to the establishment of a systematic and sustainable monitoring system for PAH contamination in foods.

## 2. Materials and Methods

### 2.1. Chemicals and Materials

The 4PAH standards, including BaA (CAS No. 56-55-3), CHR (CAS No. 218-01-9), BbF (CAS No. 205-99-2), and BaP (CAS No. 50-32-8), as well as the internal standards CHR-d_12_ (CAS No. 1719-03-5) and BaP-d_12_ (CAS No. 63466-71-7), were purchased from Sigma-Aldrich (St. Louis, MO, USA). Water (CAS No. 7732-18-5), dichloromethane (DCM; CAS No. 75-09-2), *n*-hexane (CAS No. 110-54-3), and toluene (CAS No. 108-88-3) were obtained from Honeywell International Inc. (Charlotte, NC, USA). ACN (CAS No. 75-05-8) was obtained from J.T. Baker (Phillipsburg, NJ, USA). Ethyl alcohol (CAS No. 64-17-5) and potassium hydroxide (KOH; CAS No. 1310-58-3) were provided by Samchun Pure Chemical Co., Ltd. (Pyeongtaek, Republic of Korea). *N,N*-dimethylformamide and anhydrous sodium sulfate (Na_2_SO_4_; CAS No. 7757-82-6) were sourced from Daejung Chemicals & Metals Co., Ltd. (Si-heung, Republic of Korea). Sep-Pak Florisil cartridges for solid-phase extraction (SPE) were purchased from Waters Corp. (Milford, MA, USA). The 0.45 μm PTFE membrane filters were obtained from Agilent Technologies (Santa Clara, CA, USA). QuEChERS extraction salt packets (P/N 5982–7650) containing magnesium sulfate (MgSO_4_, 4 g), sodium chloride (NaCl, 1 g), disodium citrate sesquihydrate (0.5 g), and trisodium citrate (1 g) were obtained from Agilent Technologies.

### 2.2. Sample Preparation

Sample preparation for PAH analysis involved two different extraction methods: QuEChERS and LLE. Both methods were conducted before the clean-up step. After extracting the 4PAHs, SPE (Sep-Pak Florisil cartridges) was performed for sample purification. Each method used 2 g of homogenized sample. Prior to extraction, 100 µg/kg of CHR-d_12_ and BaP-d_12_ were added as internal standards.

#### 2.2.1. Extraction Method Using QuEChERS (EN Method)

The QuEChERS method was used for sample preparation of the 4PAHs [31]. The conventional QuEChERS method usually extracts the target sample using ACN. However, to extract the 4PAHs, the solvent was replaced with HA as the extraction solvent. The preparation of HA was based on a previously published method, with additional modifications made in this study [32]. To prepare HA, 100 mL each of *n*-hexane and ACN was added to a separatory funnel and shaken vigorously. After phase separation, the lower layer of HA was collected and used. In addition, two additional solvents were prepared by adding toluene at 1% and 5% (*v*/*v*), respectively, to the prepared HA. Therefore, three types of solvents were selected to improve the extraction of PAHs: HA, 1% toluene added to HA (1T), and 5% toluene added to HA (5T).

The homogenized sample was placed in a 50 mL conical tube, followed by the addition of 5 mL of water and 10 mL of suitable extraction solvent (HA, 1T, 5T). The mixture was vortexed thoroughly, and a QuEChERS salt mix (MgSO_4_ 4 g, NaCl 1 g, disodium citrate sesquihydrate 0.5 g, trisodium citrate 1 g) was then added. The conical tube was shaken vertically using a multitube vertical mixer at 2000 rpm for 1 min and centrifuged at 4000 rpm for 5 min.

To compare the extraction of 4PAHs, the samples were divided into two groups: one was subjected to the Fo procedure, and the other was not. For the Fo procedure samples, the conical tube was frozen at −40 °C for 1 h to remove fat, as this temperature is well below the freezing point of lipids and ensures complete solidification within this time frame. Then, 5 mL of the extraction solvent layer was collected and evaporated to dryness under a gentle stream of nitrogen at 37 °C. The residue was reconstituted in 2 mL of *n*-hexane before SPE clean-up.

#### 2.2.2. Extraction Method Using LLE

The general test protocol established by the MFDS of Korea was used as the LLE method [21]. For solid samples, the sample was placed into a 300 mL round-bottom flask. For alkaline hydrolysis and enhanced release of PAHs from the sample matrix, 100 mL of 1 M KOH in ethanol was added. The mixture was refluxed at 80 °C for 3 h. After allowing for the solution to cool to room temperature, 50 mL of *n*-hexane and 50 mL of an ethanol/*n*-hexane (1:1, *v*/*v*) mixture was added. The solution was then filtered and transferred to a separatory funnel, followed by two successive extractions with 50 mL of *n*-hexane. The combined organic fractions were washed three times with 50 mL of distilled water. The resulting hexane phase was collected and dried over Na_2_SO_4_, then transferred into a 250 mL round-bottom flask and concentrated to approximately 2 mL using a rotary evaporator at 40 °C.

For non-fatty liquid (NFL) samples, the sample was placed into a 300 mL round-bottom flask, and 100 mL of *n*-hexane was added. Ultrasonic extraction was performed for 1 h. After extraction, the resulting hexane layer was collected, dried, and concentrated as described above for solid samples.

For fatty liquid (FL) samples, the sample was placed into a separatory funnel, and 100 mL of *n*-hexane and 50 mL of *N,N*-dimethylformamide/water (9:1, *v*/*v*) were added and vigorously shaken. After phase separation, the lower layer was transferred to a second separatory funnel, and the extraction was repeated twice more for a total of three extractions. Next, 100 mL of 1% (*w*/*v*) Na_2_SO_4_ solution and 50 mL of *n*-hexane were added to the separatory funnel; after phase separation, the hexane layer was transferred into a clean funnel and re-extracted twice with 35 mL portions of *n*-hexane. The combined hexane fractions were washed with 50 mL of distilled water. Subsequent drying and concentrating steps were performed as described above for the solid samples.

#### 2.2.3. Clean-Up Method Using SPE Cartridge

To eliminate interfering substances present in food, SPE was carried out following extraction using either the QuEChERS (EN method, Section 2.2.1) or the LLE method (Section 2.2.2), as previously described. SPE was performed using Sep-Pak Florisil cartridges (6 cc Vac Cartridge, 50–200 µm) as an additional purification step. The cartridges were activated with 10 mL of DCM, followed by equilibration with 20 mL of *n*-hexane. Subsequently, 2 mL of the sample extract was loaded onto the cartridge and eluted stepwise with 5 mL of *n*-hexane, followed by 15 mL of an *n*-hexane/DCM (3:1, *v*/*v*) mixture. All fractions from the sample-loading step through to the final elution were collected in a glass test tube. The collected eluates were evaporated to dryness under a gentle nitrogen stream at 37 °C. The dried residue was reconstituted in 1 mL of DCM, filtered through a 0.45 µm PTFE membrane filter, and transferred to 2 mL amber vials. An aliquot of 1 µL was injected into a gas chromatography–mass spectrometry (GC–MS) system for quantitative analysis (Section 2.3).

### 2.3. Determination of PAH Using GC-MS

PAH analysis was performed using a GC-MS system (7820A GC coupled with a 5975 MSD; Agilent Technologies). Chromatographic separation was carried out on a Zebron ZB-PAH-Select column (30 m × 0.25 mm i.d., 0.20 µm film thickness; Phenomenex, Torrance, CA, USA), specifically engineered for efficient resolution of PAHs.

A 1.0 µL aliquot of each extract was injected into the system via splitless injection mode at 320 °C to ensure efficient transfer of analytes without thermal decomposition. The initial oven temperature was held at 80 °C for 1 min, then raised to 245 °C at 6 °C/min to resolve low-molecular-weight PAHs. This was followed by a rapid temperature increase to 270 °C at 30 °C/min, maintained for 13 min to achieve the full elution of higher-molecular-weight targets. A final post-run hold at 310 °C for 10 min was applied to remove any residual analytes and recondition the column.

Helium (99.99% purity) served as the carrier gas at a constant flow rate of 1.2 mL/min. The mass spectrometer operated in electron ionization (EI) mode, utilizing selected ion monitoring (SIM) for enhanced sensitivity and selectivity toward specific PAH markers. The ion source and quadrupole temperatures were set to 250 and 150 °C, respectively, to optimize ionization efficiency and spectral stability. For CHR and BaA, the selected mass-to-charge ratios (*m/z*) were 228, 226, and 229, while *m/z* 252, 250, and 253 were used for BaP and BbF. The ions monitored for the internal standards were *m/z* 240, 236, and 241 for CHR-d_12_, and *m/z* 264, 263, and 265 for BaP-d_12_.

### 2.4. Method Validation

Validation was conducted based on the standard method performance requirements established by the criteria of the Association of Official Analytical Chemists (AOAC) [33,34]. Our representative matrices were selected according to physical characteristics and fat content, including a non-fatty solid (NFS), a fatty solid (FS), a non-fatty liquid (NFL), and a fatty liquid (FL). Fatty samples were defined as those containing ≥3% fat and non-fatty samples as those containing <3% fat. White rice, smoked pork, orange juice, and soybean oil were used as the respective representative samples. These matrices were chosen to be clean and free of PAHs, and were representative of high-consumption and frequently consumed foods in the Korean diet [35,36,37,38]. Each matrix was evaluated for selectivity, linearity, limit of detection (LOD), limit of quantification (LOQ), matrix effect (ME), accuracy, and precision.

Selectivity was assessed by spiking each matrix with standard compounds, then evaluating the presence of the target peaks and any potential interferences. Linearity was evaluated from matrix-matched calibration curves by calculating the coefficient of determination (R^2^) across five concentrations (1, 2, 5, 10, and 20 µg/kg). Each calibration curve was constructed by performing three replicate injections of 1.0 µL for every standard mixture. LOD and LOQ were calculated based on the standard deviation of the y-intercepts (SD) and the slope of the calibration curve (*S*) using the following equations:

LOD = 3.3 × SD/*S*,

LOQ = 10 × SD/*S*.

The ME was evaluated by comparing the slope of the calibration curve obtained from each sample matrix with that derived from the corresponding standard solution prepared in a blank solvent. ME was calculated using the following equation:

ME (%) = (*S* of the matrix-matched calibration curve/

*S* of the blank matrix calibration curve) × 100.

Accuracy and precision were determined by repeatedly analyzing spiked samples. Intra-day performance was assessed using three replicates within a single day, while inter-day variation was evaluated over three consecutive days.

### 2.5. Application to Commercial Samples

Monitoring was conducted on food products commercially available in South Korean retail markets to determine the levels of 4PAHs. We selected the Level-1 hierarchy of the EFSA FoodEx2 classification system; accordingly, the samples were assigned to 12 food categories [39]. A total of 74 food items were selected for analysis. For each item, 2–5 individual samples were collected, resulting in a total of 302 samples. Edible portions were homogenized and stored at −20 °C until the sample preparation process, for a maximum of 2 weeks, to ensure PAH stability. Prior to sample preparation, all samples were fully thawed at 25 °C. Sample preparation was performed according to the protocol developed in this study, and final quantification was carried out using GC-MS.

### 2.6. Risk Assessment

A risk assessment was conducted based on the U.S. Environmental Protection Agency (EPA) using toxic equivalency factors (TEFs) to estimate the relative toxicity of PAHs, where the TEF values were 1 for BaP, 0.1 for BaA, 0.01 for CHR, and 0.1 for BbF [40]. The toxic equivalency quotient (TEQ) of BaP (TEQ_BaP_) was calculated for each sample using the following equation:

TEQ_BaP_ = [*C_i_*] × TEF*_i_*,

where *C_i_* represents the concentration of each PAH congener (*i*) in the sample and TEF*_i_* refers to the toxic equivalency factor of that congener relative to the carcinogenic potency of BaP, as reported in published data. To calculate the daily intake of PAHs from the consumption of analyzed food samples, the estimated daily intake (EDI) was derived based on the TEQ_BaP_ values of each sample.

The EDI reflects the quantity of toxic PAHs consumed per kilogram of body weight per day, and was calculated using the following equation:

EDI (ng/kg bw per day) = *C* × *IR*/*BW*,

where *C_i_* is the PAH concentration in the sample, *IR* refers to the consumption rate per meal (kg), and *BW* stands for body weight (kg). The ingestion rate (*IR*) for each food was based on data from the Korea National Health and Nutrition Examination Survey (KNHANES), and the *BW* was set to the average body weight (64.5 kg) of Korean adults [41,42].

In the following equation, for evaluating the potential carcinogenic risk related to PAH exposure, the margin-of-exposure (MOE), for which the threshold value of 10,000 includes uncertainty factors, was calculated by dividing the benchmark dose lower confidence limit for a 10% response (BMDL_10_) value by the estimated daily exposure (EDI). According to EFSA, an MOE value of 10,000 or higher is generally considered to indicate a low level of concern for public health, whereas a value below this threshold may indicate potential health concerns requiring risk management actions. The BMDL_10_ value was set by the dose–response analysis for tumor type, as recommended by EFSA [43].

MOE = BMDL_10_/EDI.

The BMDL_10_ values for BaP and Σ4PAHs have been reported as 0.07 and 0.34 mg/kg bw/day, respectively [13]. Therefore, these values were conservatively adopted for the BMDL_10_ of BaP and the Σ4PAHs.

### 2.7. Statistical Analysis

All measurements were conducted in triplicate, and the results are expressed as mean values ± SD. Statistical significance was assessed at a 95% confidence level (*p* < 0.05) using one-way ANOVA, followed by Duncan’s multiple range test. Data analysis was performed using IBM SPSS Statistics software (version 27; IBM Corp., Armonk, NY, USA).

## 3. Results and Discussion

### 3.1. Method Development

#### 3.1.1. Optimization of Solvent Through Four Matrices Using QuEChERS

**Figure 1:**
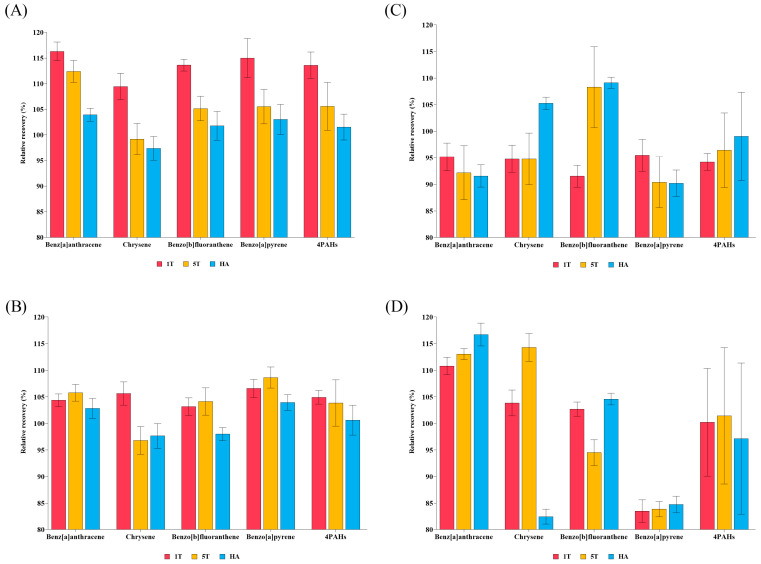
Recovery of 4PAHs by solvent type across different food matrices: (**A**) non-fatty solid, (**B**) non-fatty liquid, (**C**) fatty solid, (**D**) fatty liquid.

The recoveries of 4PAHs according to different solvents are presented in Figure 1. In NFS samples under the HA condition, BaA, CHR, BbF, and BaP were recovered at 103.95%, 97.39%, 101.78%, and 103.04%, respectively. The SDs ranged from 1.27% to 2.93%. In FS samples under the 1T condition, recoveries of BaA, CHR, BbF, and BaP were 95.16%, 94.80%, 91.53%, and 95.45%, respectively. The SDs ranged from 2.06% to 3.00%. In NFL samples under the HA condition, BaA, CHR, BbF, and BaP were recovered at 102.86%, 97.66%, 98.00%, and 103.95%, respectively. The SDs ranged from 1.24% to 2.31%. In FL samples under the 1T condition, BaA, CHR, BbF, and BaP were recovered at 110.83%, 103.87%, 102.70%, and 83.47%, respectively. The SDs ranged from 1.34% to 2.43%. Based on these recovery results, the HA solvent was most suitable for NFS and NFL matrices; in contrast, the 1T solvent showed better performance for FS and FL matrices. A previous study reported the recoveries of 4PAHs in barley, an NFS sample, as 96%, 105%, 88%, and 95% for BaA, CHR, BbF, and BaP, respectively. The SDs ranged from 2.7% to 6.3% [27]. Compared to that study, in which the deviations from 100% recovery of BaA, CHR, BbF, and BaP were −4%, +5%, −12%, and −5%, respectively, the present study shows smaller deviations of approximately +4%, −3%, +2%, and +3%, respectively, also for an NFS sample.

A previous study reported the recoveries of 4PAHs in juice, an NFL sample, as approximately 97%, 98%, 101%, and 102% for BaA, CHR, BbF, and BaP, respectively [44]. The SD ranged from 8.0% to 10.3%. The deviations from 100% recovery were approximately −3%, −2%, +1%, and +2%, for BaA, CHR, BbF, and BaP, respectively, in that study, but around +3%, −2%, −2%, and +4%, respectively, also for an NFL sample, in the present study. The afore-mentioned previous study reported larger SDs compared to those in our study, which range from 1.24% to 2.31%, indicating higher reliability. This study employed HA solvent, differing from previous studies. As 4PAHs are non-polar compounds, they tend to interact more strongly with the non-polar solvent *n*-hexane than with the polar solvent ACN. According to a previous study, partitioning measurements have demonstrated that PAHs preferentially migrate toward the *n*-hexane phase in an *n*-hexane–acetonitrile system, indicating a greater affinity for the non-polar phase [45]. Therefore, HA is expected to enhance the solubility of 4PAHs, thereby improving extraction efficiency compared to a previous study [46].

#### 3.1.2. Effect of Fo Preparation After QuEChERS Method Through Four Matrices

**Figure 2:**
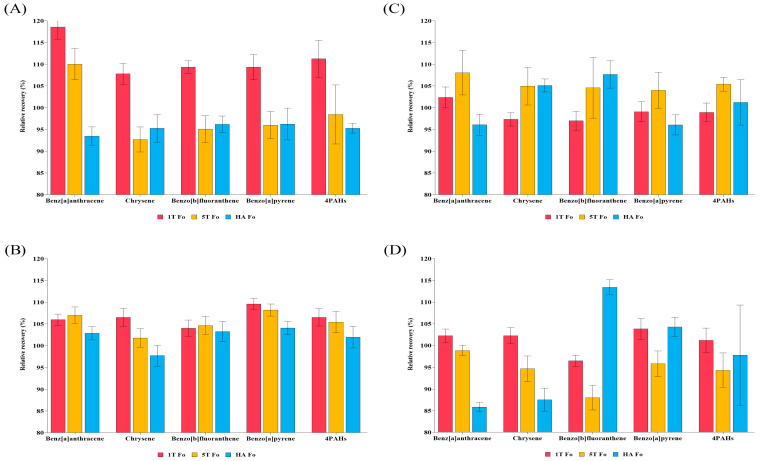
Recovery of 4PAHs by solvent type with freeze-out treatment in (**A**) non-fatty solid, (**B**) non-fatty liquid, (**C**) fatty solid, (**D**) fatty liquid.

The recoveries of 4PAHs in samples subjected to QuEChERS extraction, followed by Fo preparation, are presented in Figure 2. In NFS samples under the HA Fo condition, BaA, CHR, BbF, and BaP were recovered at 93.47%, 95.26%, 96.21%, and 96.25%, respectively. The SDs ranged from 1.88% to 3.65%. In FS samples under the 1T Fo condition, recoveries of BaA, CHR, BbF, and BaP were 102.37%, 97.32%, 97.13%, and 99.08%, respectively. The SDs ranged from 1.54% to 2.40%. In NFL samples under the HA Fo condition, BaA, CHR, BbF, and BaP were recovered at 102.85%, 97.71%, 103.25%, and 104.05%, respectively. The SDs ranged from 1.53% to 2.41%. In FL samples under the 1T Fo condition, BaA, CHR, BbF, and BaP were recovered at 102.23%, 102.27%, 96.49%, and 103.83%, respectively. The SDs ranged from 1.26% to 2.41%.

Recoveries of PAHs in ham, an FS sample, were reported in a previous study to range from approximately 72% to 111%, with a SD below 10% [6]. In the present study, the use of Fo preparation combined with 1T solvent improved recoveries to 97.13–102.37% and reduced SD to 1.54–2.40% compared to the aforementioned prior report [6]. This improvement in recovery is attributed to the addition of 1T, a non-polar aromatic solvent, which likely enhanced extraction efficiency through interactions with lipids and BaP in the meat matrix. We hypothesize, based on our experimental observations, that toluene may have contributed to increased recoveries by inducing π–π interactions or disrupting stacking between the sample matrix and PAHs, thereby reducing adsorption losses in solid samples. These findings are consistent with previous studies showing the stronger elution strength of toluene compared to hexane and improved PAH recoveries in serum and food samples [47,48]. However, when 5T was used, unstable baselines were observed, and an increasing trend in SD was noted. A previous study has reported that excessive amounts of toluene tend to cause the over-extraction of lipid components [49]. The recoveries of 4PAHs in canola oil, an FL sample, were reported in a previous study as 91%, 93%, 96%, and 92% for BaA, CHR, BbF, and BaP, respectively, with SDs ranging from about 1.2% to 6.4% [50]. While that study showed deviations from 100% recovery of approximately −9%, −7%, −4%, and −8% for BaA, CHR, BbF, and BaP, respectively, the present study demonstrated smaller deviations of 2.23%, 2.27%, 3.51%, and 3.83%, respectively, also for an FL sample. FL samples were particularly sensitive to Fo treatment, which improved the recoveries of 4PAHs. The improvement during the –40 °C freezing process is attributed to the formation of a white solidified fat layer on the upper phase. By excluding this fat layer and using only the supernatant for clean-up, lipid interference was minimized and extraction efficiency was enhanced.

Comparisons between non-Fo and Fo groups revealed that the optimal conditions varied depending on the matrix characteristics. Non-fatty samples (NFL, NFS) exhibited the highest extraction efficiency under the HA condition, whereas fatty samples (FL, FS) showed the most consistent results when 1T was combined with Fo preparation. To facilitate comparison with previous studies that employed the QuEChERS method for 4PAHs extraction, the recovery rates, LOD, and LOQ are summarized in Table 1.

### 3.2. Comparison of Validation Results Between QuEChERS and LLE Methods

Method validation was performed for each matrix using the extraction method with the highest recovery in the four-matrix recovery experiments. For NFS and NFL samples, the HA method showed the best performance, while the IT Fo method was most effective for FS and FL samples. Validation results of the QuEChERS method analyzed by GC-MS are presented in Table 2.

All four analytes—BaA, CHR, BbF, and BaP—exhibited excellent linearity, with R^2^ values ranging from 0.9973 to 0.9996. The LOD for 4PAHs in non-fatty matrices, such as white rice and orange juice, ranged from 0.03 to 0.13 µg/kg, and the LOQ ranged from 0.10 to 0.60 µg/kg. In fatty matrices, such as pork and soybean oil, LOD and LOQ were determined to be 0.07–0.19 and 0.20–0.57 µg/kg, respectively. MEs across the four matrices ranged from 78.6% to 189.3%, indicating both signal enhancement and suppression. Notably, BbF showed strong signal enhancement in rice (189.3%) and orange juice (148.8%), and suppression in soybean oil (78.6%) and pork (87.4%), demonstrating pronounced matrix dependency. Accordingly, matrix-matched calibration was applied for correction. Accuracy at low, medium, and high concentrations of 1, 5, and 20 µg/kg ranged from 94.8% to 114.5% (intra-day) and 92.0% to 114.6% (inter-day), and precision was 2.5–5.5% RSD (intra-day) and 2.3–5.7% RSD (inter-day), meeting the acceptance criteria of the AOAC guidelines for standard analytical methods [33].

Validation of 4PAHs was conducted to compare the optimized QuEChERS method with the widely used LLE method, which is employed by several international agencies and countries. While LLE analysis has drawbacks such as high solvent consumption, longer processing times, and higher costs, the QuEChERS method addresses these limitations. The developed sample preparation method was compared to the LLE method to verify its validity. The results are summarized in Table 3. The R^2^ values for the 4PAHs ranged from 0.9974 to 0.9998, indicating excellent linearity. The LODs for non-fatty samples, such as rice and orange juice, ranged from 0.04 to 0.19 µg/kg, with LOQs between 0.12 and 0.57 µg/kg. For fatty samples, such as pork and soybean oil, LODs and LOQs ranged from 0.10 to 0.20 µg/kg and from 0.31 to 0.60 µg/kg, respectively. MEs were observed in all four matrices, exhibiting bidirectional effects ranging from 111.5% to 175.7%. Notably, strong signal enhancements were observed for BaP (175.7%) and BbF (174.6%) in rice, and BaP showed a similar enhancement of approximately 159% in pork and soybean oil. Accordingly, matrix-matched calibration was applied to all samples across all matrices to correct for signal enhancement. Accuracy at low, medium, and high concentrations of 1, 5, and 20 µg/kg ranged from 95.1% to 114.7%, and inter-day accuracy ranged from 96.3% to 114.3%. Precision was within 2.3–5.5% RSD (intra-day) and 2.1–5.7% RSD (inter-day), satisfying the acceptance criteria of the AOAC guidelines for standard analytical methods [33].

When comparing the conventional LLE method with the QuEChERS GC-MS method developed in this study, the QuEChERS method showed lower LOD in most cases. Exceptions were BaA in the four matrices and CHR in the FS matrix. BaP and BbF exhibited consistently lower LOD across all matrices. This indicates that reliable quantification can be achieved with a streamlined protocol involving fewer extraction/partitioning steps, reduced handling, shorter overall preparation time, and lower solvent use. Accordingly, these results support the QuEChERS method as a simpler yet sensitive alternative to the conventional LLE method.

### 3.3. Application of the Developed Method to 302 Food Samples Distributed in Korea

This study assessed the contamination levels of PAHs in foods distributed domestically by analyzing 302 samples across 74 food items for 4PAHs. All samples were categorized according to the Level-1 criteria of the FoodEx2 food classification system established by the EFSA [39]. The results, including detected concentrations and detection rates, are presented in Table 4 and Table 5, respectively. Concentrations below the LOQ were considered non-detectable. Detection levels for each food category are expressed as mean ± SD, and detection rates for individual food items are also provided. Among the total 302 samples, 81 samples (26.8%) tested positive for at least one PAH. Among the 74 food items, 38 (51.4%) showed the presence of at least one PAH. BaA had the lowest detection rate at 10.6% (32/302), and increased for BaP at 15.2% (46/302), CHR at 17.5% (53/302), and BbF at 17.9% (54/302). Quantification was performed according to EFSA guidelines by evaluating the contamination level of samples based on the Σ4PAHs [13].

Among meat and fish products with relatively high detection rates, the top-five foods with the highest Σ4PAHs were *Kezuribushi* (22.02 ± 14.60 μg/kg), *Katsuobushi* (16.99 ± 11.08 μg/kg), meatballs (14.12 ± 11.45 μg/kg), smoked anchovy (10.63 ± 6.42 μg/kg), and grilled chicken feet (8.94 ± 4.40 μg/kg). These foods share the common characteristic of undergoing intense heat treatments, such as smoking, drying, and grilling. Processed fish products, such as *Katsuobushi* and *Kezuribushi*, are subjected to repeated smoking. These products tended to accumulate high levels of PAHs. In other categories, relatively high levels of the Σ4PAHs were detected in foods exposed to high-temperature processing, including coffee beans (12.65 ± 8.52 μg/kg), parsley powder (12.20 ± 7.20 μg/kg), palm oil (9.60 ± 5.46 μg/kg), pepper (9.16 ± 5.65 μg/kg), and seaweed (3.07 ± 1.53 μg/kg). In contrast, most liquid samples, excluding certain oils, milk, yogurt, coffee beverage, and vinegar, showed Σ4PAHs levels below the LOQ. All measured concentrations in the analyzed each samples were below the maximum limits established by the European Union for BaP and Σ4PAHs in the relevant food categories, indicating full compliance with Regulation (EU) 2023/915 [18].

According to a previous study, the Σ4PAHs in smoked ham and charcoal-grilled pork were reported as 15.2 and 19.9 μg/kg, respectively [61]. These findings are consistent with our results, indicating that smoking and charcoal grilling are associated with the formation of PAHs. Foods with relatively high levels in this study were cooked using similar methods. Additionally, a report by the UK Food Standards Agency indicated that the average Σ4PAHs concentration in herbs and spices was 17.76 μg/kg [62]. This value is higher than the levels found in pepper and parsley analyzed in our study. High-temperature processes, such as smoking, drying, and grilling, significantly contribute to PAH formation.

### 3.4. Health Risk Assessment of 4PAHs in Korean Foods

The results of dietary exposure and risk assessment for BaP and Σ4PAHs are summarized in Table 6. The table includes detected concentrations in each food type, as well as TEQ, EDI, and MOE values. To quantitatively assess the health risks of PAHs in processed foods distributed domestically, all concentrations were converted to TEQ_BaP_ using the TEFs proposed by the EFSA.

The TEQ_BaP_ ranged from 0 to 4.57 µg/kg, while for the sum of the four indicator compounds, Σ4PAHs (TEQ_4PAHs_), ranged from 0 to 4.86 µg BaP-eq/kg. The highest TEQ_BaP_ value was observed in grilled chicken feet (4.57 µg/kg), followed by *Katsuobushi* (2.62 µg/kg), *Kezuribushi* (2.50 µg/kg), *Gambas* (2.21 µg/kg), and smoked anchovy (2.10 µg/kg). Similarly, grilled chicken feet exhibited the highest TEQ_4PAHs_ (4.86 µg/kg), followed by *Kezuribushi* (3.34 µg/kg), *Katsuobushi* (3.32 µg/kg), smoked anchovy (2.63 µg/kg), and *Shabu-Shabu* broth (2.57 µg/kg). These results indicate that repeated smoking, direct flame exposure, and drying are associated with increased cumulative PAH-related toxicity.

To reflect the toxicological indicators in actual intake, EDI values were calculated using average adult consumption and body weight (64.5 kg) based on the KNHANES (2019–2021). The EDI for BaP ranged from 0 to 9.21 ng/kg bw/day, and, for the Σ4PAHs, the EDI ranged from 0 to 9.79 ng/kg bw/day. Foods with the highest BaP exposure included grilled chicken feet (9.21 ng/kg bw/day), *Shabu-Shabu* (4.80 ng/kg bw/day), beef stew (3.63 ng/kg bw/day), *Sundae* (Korean blood sausage) (2.26 ng/kg bw/day), and chicken nuggets (2.22 ng/kg bw/day). Generally, BaP was detected at higher levels in cooked meat samples, resulting in elevated EDI values. The EDI for the Σ4PAHs showed a similar pattern, ranging from 9.79 to 2.54 ng/kg bw/day for the same samples. Although some high-concentration spices showed elevated absolute concentrations, their low daily intake prevented them from ranking high in exposure.

The MOEs were calculated using the BMDL_10_ values of 0.07 mg/kg bw/day for BaP and 0.34 mg/kg bw/day for Σ4PAHs, as recommended by the EFSA. According to EFSA guidelines, MOEs close to or below 10,000 may indicate potential health concerns. The MOE for BaP from grilled chicken feet was 7604, falling below the threshold of 10,000 and suggesting a potential health risk. *Shabu-Shabu* broth and beef stew had MOEs of 14,583 and 19,285, respectively, approaching the threshold, but remaining within the safety margin. Korean *Sundae* and meatballs showed relatively higher MOEs of 31,000 and 33,000, respectively. The MOE for Σ4PAHs exceeded the concern threshold of 10,000 for all samples. Samples with relatively low MOEs were mostly meat and soup dishes with high daily intake, showing a correlation with higher EDI values. Meat products subjected to grilling and smoking generally showed MOEs between 10,000 and 100,000. When the frequency of direct flame or smoking and broth consumption is combined, the MOEs may decrease, suggesting a higher potential risk.

A previous study on PAHs in traditionally smoked meat products from the Baltic states reported MOEs of 9849 and 7205 for BaP and Σ4PAHs, respectively, raising concerns about consumer health and the need for management measures [63]. Similarly, this study observed a MOE of 7604 for BaP from grilled chicken feet, below the EFSA-recommended threshold of 10,000, consistent with the conclusion that smoked and grilled meats are potential risk foods. The Shabu-Shabu and beef stew in this study showed MOEs of 14,583 and 19,285, respectively, confirming that these are within the EFSA safety margin (>10,000). Although within the safety margin, broth dishes are in the relatively low MOE range, suggesting some caution with frequent or large servings of meat-based broths.

## 4. Conclusions

This study establishes a matrix-adapted, solvent-modified QuEChERS–GC-MS procedure for the reliable determination of the four EU indicator PAHs (BaA, CHR, BbF, BaP) in foods that differ greatly in fat content and physical state. By using 1T as the extraction solvent and integrating an optional Fo step, the method selectively weakened π–π stacking between carbonized surfaces and PAHs, while suppressing lipid co-extraction.

Method performance met internationally accepted requirements. Across the four representative matrices—NFS, FS, NFL, and FL—the protocol delivered mean recoveries of 93–110%, intra-/inter-day precision ≤5.7% RSD, LODs of 0.03–0.20 µg/kg, and R^2^ ≥ 0.997, thereby satisfying AOAC guidelines for accuracy, precision, and linearity. The Fo sample preparation further improved the extraction of 4PAHs from high-fat matrices by precipitating solid fat and minimizing MEs, resulting in SD values as low as 1.26–2.41%.

The procedure also offered clear practical advantages. Relative to conventional LLE, it cut organic solvent use from >300 to 10 mL of ACN and shortened the total preparation time from 6–24 h to <30 min, while maintaining or improving analytical sensitivity. These savings translate into lower cost, reduced environmental burden, and greater sample throughput. Because the solvent system is inexpensive and fully compatible with routine GC-MS platforms and the Fo step requires no specialized hardware, the protocol is easily scaled for regulatory or industrial quality-assurance laboratories.

Method robustness was demonstrated by its application to 302 retail foods spanning 12 EFSA FoodEx2 categories. Smoked and grilled meats showed the highest Σ4PAHs, reaching 22 µg/kg in *Kezuribushi*, whereas staples such as white rice remained below detection limits. MOE analysis identified grilled chicken feet with a MOE of 7604, below the health-protective threshold of 10,000, highlighting the need for targeted risk-reduction measures. Meanwhile, the majority of products remained within safe limits. The validated method, therefore, enables sensitive and scalable monitoring of PAHs and supports evidence-based food safety management.

A key limitation is that a single solvent composition and Fo setting were applied, regardless of the extent of charring. The literature and experimental observations show that strongly carbonized matrices still suffer decreased recovery, owing to irreversible adsorption on carbonaceous surfaces. The Fo step was applied under a fixed condition (−40 °C for 1 h) across all matrices without systematic optimization by charring level, which may have constrained performance. Future studies should refine the solvent strength, sorbent selection, and Fo parameters under systematically varied carbonization conditions, producing extraction protocols tailored to the degree of carbonization that secure complete desorption from heavily carbonized samples. In addition, increasing the sample number in future studies will be essential to ensuring experimental reliability and enabling a more accurate assessment of contamination risks across a wider and more diverse range of food matrices.

In conclusion, extraction with HA or 1T, followed by SPE clean-up, offers a simple, precise, solvent-saving, and time-saving route for the quantification of 4PAHs across diverse food matrices. Incorporating carbonization-aware fine-tuning will further strengthen real-world applicability, enhance regulatory enforcement, and close critical data gaps in dietary PAH exposure science.

## Figures and Tables

**Table 1:** Summary of recovery and sensitivity parameters from previous studies on QuEChERS-based analysis of four PAHs in food matrices.

Study (Year)	Food Sample	Extraction Method and Solvent	Recovery BaA (%)	Recovery CHR (%)	Recovery BbF (%)	Recovery BaP (%)	LOD BaA (µg/kg)	LOQ BaA (µg/kg)	LOD CHR (µg/kg)	LOQ CHR (µg/kg)	LOD BbF (µg/kg)	LOQ BbF (µg/kg)	LOD BaP (µg/kg)	LOQ BaP (µg/kg)	Ref.
Sun & Wu (2020)	Soybean oil	acetonitrile: acetone (3:2, *v*/*v*) QuEChERS	112.87	87.25	66.72	83.51	0.07	0.23	0.06	0.20	0.09	0.30	0.11	0.36	[51]
Ma et al. (2024)	Soybean oil	acetonitrile: acetone (3:2, *v*/*v*) QuEChERS	92.68	97.76	109.02	80.64	0.00	0.17	0.03	0.17	0.17	0.23	0.17	0.33	[52]
Prata et al. (2024)	Fish and vegetables	acetonitrile: QuEChERS	95.00	95.00	92.00	83.00	0.05	0.10	0.05	0.10	0.05	0.10	0.05	0.10	[53]
Surma et al. (2018)	Pork ham	ethyl acetate: QuEChERS	97.00	78.00	72.00	101.00	1.00	3.00	0.20	0.50	0.20	0.60	0.70	2.10	[6]
Hwang et al. (2021)	Herbal Medicine Ingredients	hexane: acetone (1:1; *v*/*v*) QuEChERS	111.22	118.59	103.50	103.79	0.12	0.37	0.17	0.51	0.14	0.41	0.08	0.25	[54]
Singh & Agarwal (2021)	Chicken	acetonitrile (1% acetic acid) QuEChERs	89.53	91.62	89.66	87.56	0.03	0.09	0.03	0.09	0.30	0.90	0.06	0.18	[8]
Ramalhosa et al. (2012)	Fish	acetonitrile: QuEChERS	87.40	90.70	95.10	86.60	0.09	0.30	0.21	0.71	0.23	0.77	0.17	0.56	[55]
Ciecierska et al. (2023)	Sausage	acetonitrile: QuEChERS	88.80	86.20	87.30	89.50	0.05	0.10	0.08	0.16	0.10	0.20	0.12	0.24	[2]
Eklu-Gadegbeku et al. (2020)	Fish	acetonitrile: QuEChERS	95.00	92.00	90.00	72.00	-	0.90	-	0.90	-	0.90	-	0.90	[15]
Diop et al. (2023)	Fish	acetonitrile: acetone (6:4 *v*/*v*) QuEChERS	-	-	-	-	0.09	0.24	0.07	0.20	0.05	0.19	0.08	0.23	[56]
Khorshid et al. (2015)	Fish	acetonitrile: QuEChERS	96.00	89.00	76.00	70.00	0.50	2.00	0.33	2.00	0.57	2.00	0.37	2.00	[57]
Jeong et al. (2025)	Egg	acetonitrile: QuEChERS	88.90	91.80	89.79	89.65	0.03	0.09	0.02	0.05	0.01	0.04	0.01	0.04	[50]
Harrison et al. (2024)	Black tea leaves	acetonitrile: QuEChERS	105.00	108.00	109.00	112.00	0.11	0.38	0.13	0.43	0.13	0.42	0.02	0.08	[58]
Al-Thaiban et al. (2018)	Beef, turkey	acetonitrile: QuEChERS	110.00	110.00	112.00	105.00	0.69	1.13	0.44	0.62	0.24	0.41	0.34	0.63	[59]
Pincemaille et al. (2014)	Tea infusions	acetonitrile: acetone (6:4 *v*/*v*) QuEChERS	84.00	88.00	67.00	72.00	0.10	0.30	0.10	0.20	0.10	0.30	0.10	0.40	[60]

**Table 2:** Method validation results for QuEChERS quantification of 4PAHs in four food matrices.

Matrix (Representative Sample)	Compound	Linearity (R^2^)	ME * (%)	Intra-Day (*n* = 3)						Inter-Day (*n* = 3)						LOD * (µg/kg)	LOQ * (µg/kg)
				Accuracy			Precision			Accuracy			Precision				
				(%)			(%RSD)			(%)			(%RSD)				
				1 µg/kg	5 µg/kg	20 µg/kg	1 µg/kg	5 µg/kg	20 µg/kg	1 µg/kg	5 µg/kg	20 µg/kg	1 µg/kg	5 µg/kg	20 µg/kg		
Non-fatty solid	BaA	0.9990	124.5	111.2	110.6	108.9	3.3	3.1	3.0	109.8	111	110.2	2.7	2.3	3.8	0.20	0.60
(white rice)	CHR	0.9985	108.0	104.6	103.8	100.2	4.3	4.4	3.9	103.7	105.1	101.5	2.6	3.8	2.5	0.03	0.10
	BbF	0.9994	189.3	103.3	104.2	108.3	3.4	3.2	3.0	101.4	103.8	109.1	4.8	3.7	2.5	0.06	0.17
	BaP	0.9980	152.4	105.6	95.3	95.1	4.1	5.2	3.0	108.2	95.9	95.8	3.0	4.1	2.7	0.04	0.11
Fatty solid	BaA	0.9992	138	112.8	112.7	114.5	2.7	2.9	4.2	112.6	114.5	114.6	2.6	3.9	4.2	0.16	0.5
(smoked Pork)	CHR	0.9978	118.9	105.1	105.6	103.4	3.9	4.8	2.5	104.7	106	103.2	3.1	4.7	4.4	0.11	0.33
	BbF	0.9987	87.4	105.0	109.3	114.2	3.1	3	5.1	104.1	109.2	114.8	3.3	3.0	3.3	0.16	0.48
	BaP	0.9973	132.0	107.1	94.8	95.8	3.0	5.3	3.5	112.1	94.0	92.0	2.8	4.3	2.6	0.13	0.39
Non-fatty liquid	BaA	0.9994	114.7	110.4	112.7	113.3	2.5	3.3	2.7	110	110.4	113	3.9	2.7	2.6	0.13	0.41
(orange juice)	CHR	0.9984	100.0	105.2	104.3	101.4	2.6	3.8	3.5	105.6	105.8	101.6	2.5	3.4	4.3	0.04	0.12
	BbF	0.9996	148.8	105.9	101.4	107.2	4.8	3.5	2.7	101.9	102.1	105.5	5.7	2.6	3.2	0.09	0.26
	BaP	0.9972	128.3	109.4	94.8	95.2	5.3	5.5	2.6	114.7	95.1	96.5	2.6	4.8	3.2	0.11	0.33
Fatty liquid	BaA	0.9994	139.2	111.5	111.2	112.1	4.5	3.7	3.9	109.8	114	112.5	3.1	3.2	3.0	0.19	0.57
(soybean oil)	CHR	0.9986	114.2	106.1	107.5	103.2	2.7	4.5	3.2	105.8	108	104.3	2.5	3.9	2.8	0.13	0.4
	BbF	0.9990	78.6	104.7	108.9	113.8	2.6	3.1	4.8	103.6	109.5	111.2	2.9	2.5	2.6	0.11	0.34
	BaP	0.9973	130.9	99.5	96.2	105.7	2.8	4.8	2.9	101.3	96.1	95.4	2.5	4.2	3.5	0.07	0.20

* LOD: limit of detection; LOQ: limit of quantitation; ME: matrix effect.

**Table 3:** Method validation results for LLE quantification of 4PAHs in four food matrices.

Matrix (Representative Sample)	Compound	Linearity (R^2^)	ME * (%)	Intra-Day (*n* = 3)						Inter-Day (*n* = 3)						LOD * (µg/kg)	LOQ * (µg/kg)
				Accuracy			Precision			Accuracy			Precision				
				(%)			(%RSD)			(%)			(%RSD)				
				1 µg/kg	5 µg/kg	20 µg/kg	1 µg/kg	5 µg/kg	20 µg/kg	1 µg/kg	5 µg/kg	20 µg/kg	1 µg/kg	5 µg/kg	20 µg/kg		
Non-fatty solid	BaA	0.9990	138.7	113.9	106.4	110.0	4.0	3.4	3.6	114.3	112.8	112.6	5.0	5.7	3.5	0.19	0.57
(white rice)	CHR	0.9995	126.3	101.2	106.0	111.6	5.0	2.5	3.1	108.0	107.2	111.3	2.6	2.9	3.7	0.14	0.41
	BbF	0.9978	174.6	104.8	110.6	97.8	2.6	3.4	3.8	113.2	110.5	98.9	5.5	3.0	2.6	0.08	0.25
	BaP	0.9998	175.7	114.7	103.1	111.7	3.8	3.7	2.4	113.5	101.8	109.3	4.9	3.4	4.5	0.04	0.12
Fatty solid	BaA	0.9995	113.6	108.7	105.0	114.7	5.5	3.0	2.7	114.3	106.2	113.7	5.2	3.7	3.5	0.12	0.36
(smoked Pork)	CHR	0.9994	115.2	111.8	113.4	109.3	2.5	2.6	4.5	113.8	114.0	109.0	3.4	2.1	2.9	0.15	0.46
	BbF	0.9990	142.7	106.0	103.9	97.5	5.5	4.4	3.6	107.5	102.7	97.6	3.0	2.9	3.3	0.17	0.52
	BaP	0.9974	159.0	112.8	115.3	109.0	3.8	2.7	2.6	111.8	112.3	107.6	5.7	4.2	2.9	0.20	0.60
Non-fatty liquid	BaA	0.9992	127.9	110.4	99.7	102.3	4.9	2.5	3.5	112.0	98.2	107.5	2.6	2.6	2.8	0.13	0.39
(orange juice)	CHR	0.9992	116.3	112.7	103.1	101.0	3.9	3.5	2.4	111.9	102.8	101.3	3.9	4.9	3.7	0.16	0.49
	BbF	0.9996	133.1	110.0	97.4	95.1	2.3	4.7	5.0	112.7	97.7	98.2	4.3	3.0	2.7	0.13	0.38
	BaP	0.9997	150.3	112.6	96.8	98.8	3.5	5.3	3.2	112.5	102.4	98.3	3.1	3.3	3.6	0.12	0.36
Fatty liquid	BaA	0.9995	115.9	108.0	104.3	113.7	3.9	3.2	3.4	113.3	103.4	113.0	5.2	4.3	3.5	0.14	0.41
(soybean oil)	CHR	0.9995	111.5	103.9	108.5	105.1	5.2	4.5	3.7	103.8	109.4	103.8	2.6	2.9	3.7	0.18	0.56
	BbF	0.9995	131.9	101.2	98.4	95.5	2.6	2.5	3.9	100.1	98.9	96.3	5.5	3	2.6	0.12	0.37
	BaP	0.9992	159.8	105.0	102.4	110.4	3.8	4.9	2.6	104.6	101.3	109.7	4.9	3.4	4.5	0.10	0.31

* LOD: limit of detection; LOQ: limit of quantitation; ME: matrix effect.

**Table 4:** Detected concentration of 4PAHs from 74 food items.

Food Categories	Food Item (Number of Samples)	Concentration* (µg/kg)				
		Benz[a]anthracene	Chrysene	Benzo[b]fluoranthene	Benzo[a]pyrene	∑4PAHs
Grain and grain -based products	Rice (4)	N·D*	N·D	N·D	N·D	N·D
	Sorghum (3)	N·D	N·D	0.29 ± 0.15	N·D	0.29 ± 0.15
	Rice cake (3)	N·D	N·D	N·D	N·D	N·D
	Noodle (5)	N·D	N·D	N·D	N·D	N·D
	Flour (5)	N·D	N·D	N·D	N·D	N·D
	Cereal (2)	N·D	N·D	N·D	N·D	N·D
Vegetables and vegetable products	Tomato (4)	N·D	N·D	N·D	N·D	N·D
	Seaweed (5)	0.75 ± 0.31	0.81 ± 0.45	1.28 ± 0.67	0.24 ± 0.10	3.07 ± 1.53
Legumes, nuts, and oilseeds	Tofu (3)	N·D	N·D	N·D	N·D	N·D
Meat and meat products	Raw chicken meat (5)	N·D	N·D	N·D	N·D	N·D
	Smoked chicken breast (4)	N·D	0.24 ± 0.12	0.21 ± 0.04	0.19 ± 0.08	0.64 ± 0.25
	Beef steak (5)	N·D	0.67 ± 0.26	0.98 ± 0.51	1.02 ± 0.45	2.67 ± 1.22
	Bulgogi (5)	1.26 ± 0.43	N·D	0.67 ± 0.31	0.75 ± 0.26	2.68 ± 0.99
	Spicy stir-fried chicken (5)	N·D	N·D	N·D	N·D	N·D
	Grilled chicken feet (5)	1.79 ± 0.74	1.64 ± 1.07	0.93 ± 0.36	4.57 ± 2.23	8.94 ± 4.40
	Meat ball (5)	5.53 ± 4.02	5.18 ± 5.21	2.37 ± 1.71	1.04 ± 0.51	14.12 ± 11.45
	Chicken nugget (5)	N·D	N·D	1.56 ± 0.58	1.10 ± 0.40	2.67 ± 0.99
Fish and other seafood	Herring Roe (3)	0.80 ± 0.38	N·D	0.14 ± 0.06	N·D	0.94 ± 0.44
	Squid (4)	N·D	0.26 ± 0.12	0.18 ± 0.08	N·D	0.45 ± 0.20
	Oyster (2)	N·D	0.96 ± 0.55	0.42 ± 0.19	0.22 ± 0.10	1.60 ± 0.84
	Jeotgal (3)	N·D	1.03 ± 0.49	0.35 ± 0.17	N·D	1.39 ± 0.65
	Braised Cutlassfish (4)	N·D	0.16 ± 0.08	N·D	0.51 ± 0.62	0.67 ± 0.70
	Fish jerky (4)	1.14 ± 0.63	1.43 ± 1.13	0.25 ± 0.28	0.49 ± 0.23	3.31 ± 2.27
	Grilled squid (5)	1.14 ± 0.46	0.14 ± 0.10	0.32 ± 0.12	0.44 ± 0.26	2.03 ± 0.94
	Fried squid (5)	N·D	0.64 ± 0.24	N·D	N·D	0.64 ± 0.24
	Grilled Mackerel (5)	N·D	N·D	N·D	N·D	N·D
	Katsuobushi (5)	3.54 ± 1.73	8.14 ± 6.12	2.69 ± 1.83	2.62 ± 1.40	16.99 ± 11.08
	Kezuribushi (5)	5.44 ± 3.95	12.37 ± 8.10	1.70 ± 0.46	2.50 ± 2.09	22.02 ± 14.60
	Dried anchovy powder (5)	N·D	N·D	N·D	N·D	N·D
	Dried anchovy (5)	N·D	N·D	N·D	N·D	N·D
	Smoked anchovy (5)	3.16 ± 2.18	3.62 ± 2.30	1.76 ± 0.82	2.10 ± 1.12	10.63 ± 6.42
	Smoked oyster (5)	0.90 ± 0.46	2.12 ± 1.09	0.60 ± 0.32	0.55 ± 0.29	4.17 ± 2.15
Milk and dairy products	Pudding (4)	N·D	N·D	N·D	N·D	N·D
	Milk (4)	N·D	N·D	N·D	N·D	N·D
	Yogurt (4)	N·D	N·D	N·D	N·D	N·D
Sugar and confectionery	Sugar (5)	N·D	N·D	N·D	N·D	N·D
	Honey (4)	N·D	N·D	N·D	N·D	N·D
	Starch syrup (4)	N·D	N·D	N·D	N·D	N·D
Fats and oils	Palm oil (3)	2.49 ± 1.38	2.16 ± 0.97	3.80 ± 2.60	1.15 ± 0.52	9.60 ± 5.46
	Olive oil (2)	N·D	0.99 ± 0.52	1.03 ± 0.56	0.89 ± 0.50	2.92 ± 1.58
	Corn oil (2)	0.68 ± 0.36	1.38 ± 0.75	0.61 ± 0.32	N·D	2.68 ± 1.43
	Perilla oil (2)	N·D	N·D	N·D	0.31 ± 0.15	0.31 ± 0.15
	Soybean oil (2)	N·D	N·D	N·D	N·D	N·D
	Sunflower oil (2)	N·D	N·D	N·D	N·D	N·D
	Walnut oil (2)	N·D	N·D	N·D	N·D	N·D
	Flavor oil (2)	N·D	N·D	N·D	0.34 ± 0.13	0.34 ± 0.13
	Shortening (3)	N·D	N·D	1.18 ± 0.73	1.50 ± 0.80	2.69 ± 1.53
	Flaxseed oil (2)	N·D	N·D	N·D	N·D	N·D
	Fish oil (2)	N·D	N·D	N·D	N·D	N·D
	Sesame oil (2)	N·D	N·D	N·D	N·D	N·D
Non-alcoholic beverages	Coffee bean (5)	4.13 ± 1.74	3.40 ± 3.68	3.09 ± 1.59	2.04 ± 1.51	12.65 ± 8.52
	Instant coffee (5)	N·D	N·D	N·D	N·D	N·D
	Coffee liquid (5)	N·D	N·D	N·D	N·D	N·D
	Tomato juice (3)	N·D	N·D	N·D	N·D	N·D
Herbs, spices and condiments	Pepper (4)	1.99 ± 1.10	4.22 ± 2.89	1.53 ± 1.05	1.42 ± 0.62	9.16 ± 5.65
	Turmeric powder (4)	N·D	0.79 ± 0.36	N·D	N·D	0.79 ± 0.36
	Parsley powder (3)	1.73 ± 0.86	6.33 ± 4.02	2.73 ± 1.71	1.41 ± 0.61	12.20 ± 7.20
	Acorn powder (5)	N·D	N·D	N·D	N·D	N·D
	Green tea powder (5)	N·D	N·D	N·D	N·D	N·D
	Red pepper paste (5)	N·D	N·D	N·D	N·D	N·D
	Pork cutlet sauce (4)	N·D	N·D	2.41 ± 1.03	0.78 ± 0.35	3.20 ± 1.38
	Chili Sauce (4)	N·D	N·D	N·D	0.88 ± 0.40	0.88 ± 0.40
	Mayonnaise (4)	N·D	N·D	N·D	N·D	N·D
	Tsuyu sauce (5)	N·D	N·D	N·D	N·D	N·D
	Soy sauce (5)	N·D	N·D	N·D	N·D	N·D
	Vinegar (4)	N·D	N·D	N·D	N·D	N·D
Composite food	Gambas (5)	1.06 ± 0.46	1.26 ± 0.53	N·D	2.21 ± 0.96	4.52 ± 1.95
	Seafood stew (5)	N·D	0.34 ± 0.13	1.04 ± 0.70	0.45 ± 0.35	1.82 ± 1.18
	Beef stew (5)	N·D	0.52 ± 0.43	1.42 ± 1.20	1.53 ± 0.69	3.47 ± 2.32
	Beef seaweed stew (5)	N·D	N·D	N·D	1.12 ± 0.49	1.12 ± 0.49
	Shabu-shabu (5)	1.28 ± 0.53	N·D	4.16 ± 1.70	2.03 ± 0.85	7.46 ± 3.08
	Spicy Korean rice cake (5)	N·D	N·D	N·D	N·D	N·D
	Korean blood sausage (5)	N·D	N·D	N·D	1.12 ± 0.55	1.12 ± 0.55
Snacks, desserts, and other foods	Cacao nibs (3)	N·D	N·D	N·D	N·D	N·D

* Concentration (mean ± standard deviation); N⋅D: Not Detected.

**Table 5:** Detection rate of 4PAHs from 74 food items.

Food Categories	Food Item (Number of Samples)	Detection Rate (%)			
		Benz[a]anthracene	Chrysene	Benzo[b]fluoranthene	Benzo[a]pyrene
Grain and grain -based products	Rice (4)	0.0	0.0	0.0	0.0
	Sorghum (3)	0.0	0.0	33.3	0.0
	Rice cake (3)	0.0	0.0	0.0	0.0
	Noodle (5)	0.0	0.0	0.0	0.0
	Flour (5)	0.0	0.0	0.0	0.0
	Cereal (2)	0.0	0.0	0.0	0.0
Vegetables and vegetable products	Tomato (4)	0.0	0.0	0.0	0.0
	Seaweed (5)	20.0	40.0	40.0	20.0
Legumes, nuts, and oilseeds	Tofu (3)	0.0	0.0	0.0	0.0
Meat and meat products	Raw chicken meat (5)	0.0	0.0	0.0	0.0
	Smoked chicken breast (4)	0.0	75.0	25.0	50.0
	Beef steak (5)	0.0	20.0	40.0	20.0
	Bulgogi (5)	20.0	0.0	40.0	20.0
	Spicy stir-fried chicken (5)	0.0	0.0	0.0	0.0
	Grilled chicken feet (5)	20.0	40.0	20.0	20.0
	Meat ball (5)	40.0	60.0	60.0	40.0
	Chicken nugget (5)	0.0	0.0	20.0	20.0
Fish and other seafood	Herring Roe (3)	33.3	0.0	33.3	0.0
	Squid (4)	0.0	25.0	25.0	0.0
	Oyster (2)	0.0	50.0	50.0	50.0
	Jeotgal (3)	0.0	33.3	33.3	0.0
	Braised Cutlassfish (4)	0.0	50.0	0.0	50.0
	Fish jerky (4)	50.0	75.0	100.0	25.0
	Grilled squid (5)	20.0	40.0	20.0	40.0
	Fried squid (5)	0.0	20.0	0.0	0.0
	Grilled Mackerel (5)	0.0	0.0	0.0	0.0
	Katsuobushi (5)	100.0	100.0	80.0	60.0
	Kezuribushi (5)	100.0	100.0	100.0	80.0
	Dried anchovy powder (5)	0.0	0.0	0.0	0.0
	Dried anchovy (5)	0.0	0.0	0.0	0.0
	Smoked anchovy (5)	60.0	60.0	60.0	60.0
	Smoked oyster (5)	40.0	60.0	40.0	
Milk and dairy products	Pudding (4)	0.0	0.0	0.0	0.0
	Milk (4)	0.0	0.0	0.0	0.0
	Yogurt (4)	0.0	0.0	0.0	0.0
Sugar and confectionery	Sugar (5)	0.0	0.0	0.0	0.0
	Honey (4)	0.0	0.0	0.0	0.0
	Starch syrup (4)	0.0	0.0	0.0	0.0
Fats and oils	Palm oil (3)	33.3	33.3	100.0	33.3
	Olive oil (2)	0.0	50.0	50.0	50.0
	Corn oil (2)	50.0	50.0	50.0	0.0
	Perilla oil (2)	0.0	0.0	0.0	50.0
	Soybean oil (2)	0.0	0.0	0.0	0.0
	Sunflower oil (2)	0.0	0.0	0.0	0.0
	Walnut oil (2)	0.0	0.0	0.0	0.0
	Flavor oil (2)	0.0	0.0	0.0	100.0
	Shortening (3)	0.0	0.0	66.7	33.3
	Flaxseed oil (2)	0.0	0.0	0.0	0.0
	Fish oil (2)	0.0	0.0	0.0	0.0
	Sesame oil (2)	0.0	0.0	0.0	0.0
Non-alcoholic beverages	Coffee bean (5)	20.0	60.0	40.0	60.0
	Instant coffee (5)	0.0	0.0	0.0	0.0
	Coffee liquid (5)	0.0	0.0	0.0	0.0
	Tomato juice (3)	0.0	0.0	0.0	0.0
Herbs, spices and condiments	Pepper (4)	50.0	75.0	50.0	25.0
	Turmeric powder (4)	0.0	25.0	0.0	0.0
	Parsley powder (3)	33.3	33.3	33.3	33.3
	Acorn powder (5)	0.0	0.0	0.0	0.0
	Green tea powder (5)	0.0	0.0	0.0	0.0
	Red pepper paste (5)	0.0	0.0	0.0	0.0
	Pork cutlet sauce (4)	0.0	0.0	25.0	25.0
	Chili Sauce (4)	0.0	0.0	0.0	25.0
	Mayonnaise (4)	0.0	0.0	0.0	0.0
	Tsuyu sauce (5)	0.0	0.0	0.0	0.0
	Soy sauce (5)	0.0	0.0	0.0	0.0
	Vinegar (4)	0.0	0.0	0.0	0.0
Composite food	Gambas (5)	20.0	20.0	0.0	20.0
	Seafood stew (5)	0.0	20.0	40.0	40.0
	Beef stew (5)	0.0	40.0	40.0	20.0
	Beef seaweed stew (5)	0.0	0.0	0.0	20.0
	Shabu-shabu (5)	20.0	0.0	20.0	20.0
	Spicy Korean rice cake (5)	0.0	0.0	0.0	0.0
	Korean blood sausage (5)	0.0	0.0	0.0	40.0
Snacks, desserts, and other foods	Cacao nibs (3)	0.0	0.0	0.0	0.0

**Table 6:** Health risk assessment of the 4PAHs in commercially available foods in Korea.

Food Item	BaP Con. * (µg/kg)	TEQ_BaP_ (µg/kg)	EDI_BaP_ (ng/kg b.w/day)	BaP MOEs	4PAHs Con. (µg/kg)	TEQ_4PAHs_ (µg BaP-eq/kg)	EDI_4PAHs_ (ng/kg b.w/day)	4PAHs MOEs
Rice	N·D	0.00	0.00	-	N·D	0.00	0.00	-
Sorghum	N·D	0.00	0.00	-	0.2944	0.03	0.13	2,582,888
Rice cake	N·D	0.00	0.00	-	N·D	0.00	0.00	-
Noodle	N·D	0.00	0.00	-	N·D	0.00	0.00	-
Flour	N·D	0.00	0.00	-	N·D	0.00	0.00	-
Cereal	N·D	0.00	0.00	-	N·D	0.00	0.00	-
Tomato	N·D	0.00	0.00	-	N·D	0.00	0.00	-
Seaweed	0.2414	0.24	0.01	5,343,828	3.0702	0.45	0.02	13,874,324
Tofu	N·D	0.00	0.00	-	N·D	0.00	0.00	-
Raw chicken meat	N·D	0.00	0.00	-	N·D	0.00	0.00	-
Smoked chicken breast	0.1947	0.19	0.39	178,656	0.6403	0.22	0.44	775,131
Beef steak	1.0182	1.02	2.05	34,163	2.6680	1.12	2.26	150,488
Bulgogi	0.7469	0.75	1.50	46,543	2.6800	0.94	1.89	179,586
Spicy stir-fried chicken	N·D	0.00	0.00	-	N·D	0.00	0.00	-
Grilled chicken feet	4.5744	4.57	9.21	7604	8.9432	4.86	9.79	34,739
Meat ball	1.0402	1.04	2.09	33,440	14.1238	1.88	3.79	89,777
Chicken nugget	1.1039	1.10	2.22	31,510	2.6657	1.26	2.54	134,081
Herring Roe	N·D	0.00	0.00	-	0.9419	0.09	0.06	5,255,694
Squid	N·D	0.00	0.00	-	0.4470	0.02	0.01	23,459,097
Oyster	0.2241	0.22	0.15	454,791	1.5969	0.28	0.19	1,798,194
Jeotgal	N·D	0.00	0.00	-	1.3879	0.05	0.03	11,489,183
Braised Cutlassfish	0.5059	0.51	0.32	222,007	0.6691	0.51	0.32	1,074,853
Fish jerky	0.4918	0.49	0.34	207,236	3.3060	0.64	0.44	767,958
Grilled squid	0.4363	0.44	0.30	233,598	2.0323	0.58	0.40	848,979
Fried squid	N·D	0.00	0.00	-	0.6403	0.01	0.00	85,197,937
Grilled Mackerel	N·D	0.00	0.00	-	N·D	0.00	0.00	-
Katsuobushi	2.6191	2.62	1.80	38,914	16.9934	3.32	2.28	148,921
Kezuribushi	2.5048	2.50	1.72	40,690	22.0180	3.34	2.30	148,103
Dried anchovy powder	N·D	0.00	0.00	-	N·D	0.00	0.00	-
Dried anchovy	N·D	0.00	0.00	-	N·D	0.00	0.00	-
Smoked anchovy	2.1006	2.10	1.44	48,519	10.6324	2.63	1.81	188,339
Smoked oyster	0.5531	0.55	0.34	203,062	4.1663	0.72	0.45	753,711
Pudding	N·D	0.00	0.00	-	N·D	0.00	0.00	-
Milk	N·D	0.00	0.00	-	N·D	0.00	0.00	-
Yogurt	N·D	0.00	0.00	-	N·D	0.00	0.00	-
Sugar	N·D	0.00	0.00	-	N·D	0.00	0.00	-
Honey	N·D	0.00	0.00	-	N·D	0.00	0.00	-
Starch syrup	N·D	0.00	0.00	-	N·D	0.00	0.00	-
Palm oil	1.1545	1.15	0.14	507,894	9.5969	1.80	0.22	1,578,238
Olive oil	0.8945	0.89	0.11	655,521	2.9191	1.01	0.12	2,826,144
Corn oil	N·D	0.00	0.00	-	2.6755	0.14	0.02	19,929,129
Perilla oil	0.3147	0.31	0.04	1,863,246	0.3147	0.31	0.04	9,050,054
Soybean oil	N·D	0.00	0.00	-	N·D	0.00	0.00	-
Sunflower oil	N·D	0.00	0.00	-	N·D	0.00	0.00	-
Walnut oil	N·D	0.00	0.00	-	N·D	0.00	0.00	-
Flavor oil	0.3375	0.34	0.04	1,737,631	0.3375	0.34	0.04	8,439,923
Shortening	1.5003	1.50	0.18	390,831	2.6851	1.62	0.19	1,759,387
Flaxseed oil	N·D	0.00	0.00	-	N·D	0.00	0.00	-
Fish oil	N·D	0.00	0.00	-	N·D	0.00	0.00	-
Sesame oil	N·D	0.00	0.00	-	N·D	0.00	0.00	-
Instant coffee	N·D	0.00	0.00	-	N·D	0.00	0.00	-
Coffee liquid	N·D	0.00	0.00	-	N·D	0.00	0.00	-
Tomato juice	N·D	0.00	0.00	-	N·D	0.00	0.00	-
Pepper	1.4220	1.42	0.82	85,629	9.1605	1.82	1.04	325,702
Turmeric powder	N·D	0.00	0.00	-	0.7880	0.01	0.00	75,053,801
Parsley powder	1.4100	1.41	0.81	86,357	12.1950	1.92	1.10	308,180
Acorn powder	N·D	0.00	0.00	-	N·D	0.00	0.00	-
Green tea powder	N·D	0.00	0.00	-	N·D	0.00	0.00	-
Red pepper paste	N·D	0.00	0.00	-	N·D	0.00	0.00	-
Pork cutlet sauce	0.7825	0.78	0.05	1,407,309	3.1963	1.02	0.07	5,224,031
Chili Sauce	0.8843	0.88	0.06	1,245,301	0.8843	0.88	0.06	6,048,604
Mayonnaise	N·D	0.00	0.00	-	N·D	0.00	0.00	-
Tsuyu sauce	N·D	0.00	0.00	-	N·D	0.00	0.00	-
Soy sauce	N·D	0.00	0.00	-	N·D	0.00	0.00	-
Vinegar	N·D	0.00	0.00	-	N·D	0.00	0.00	-
Gambas	2.2077	2.21	1.52	46,165	4.5233	2.33	1.60	212,810
Seafood stew	0.4470	0.45	1.06	66,065	1.8243	0.55	1.31	258,648
Beef stew	1.5313	1.53	3.63	19,285	3.4706	1.68	3.98	85,474
Beef seaweed stew	1.1166	1.12	2.65	26,447	1.1166	1.12	2.65	128,458
Shabu-shabu	2.0251	2.03	4.80	14,583	7.4645	2.57	6.09	55,833
Spicy Korean rice cake	N·D	0.00	0.00	-	N·D	0.00	0.00	-
Korean blood sausage	1.1214	1.12	2.26	31,019	1.1214	1.12	2.26	150,662
Cacao nibs	N·D	0.00	0.00	-	N·D	0.00	0.00	-

* Con.: Concentration.

## Data Availability

The original contributions presented in this study are included in the article. Further inquiries can be directed to the corresponding author.
